# Can Australian meal kits support food literacy and healthy family meal provisioning? A qualitative study

**DOI:** 10.1093/heapro/daaf105

**Published:** 2025-07-08

**Authors:** Kylie Fraser, Penelope Love, Karen J Campbell, Alison Spence

**Affiliations:** Institute for Physical Activity and Nutrition (IPAN), School of Exercise & Nutrition Sciences, Deakin University, 75 Pigdons Road, Waurn Ponds, Djilang Geelong, Victoria 3216, Australia; Institute for Physical Activity and Nutrition (IPAN), School of Exercise & Nutrition Sciences, Deakin University, 75 Pigdons Road, Waurn Ponds, Djilang Geelong, Victoria 3216, Australia; Institute for Physical Activity and Nutrition (IPAN), School of Exercise & Nutrition Sciences, Deakin University, 75 Pigdons Road, Waurn Ponds, Djilang Geelong, Victoria 3216, Australia; Institute for Physical Activity and Nutrition (IPAN), School of Exercise & Nutrition Sciences, Deakin University, 75 Pigdons Road, Waurn Ponds, Djilang Geelong, Victoria 3216, Australia

**Keywords:** food literacy, meal kits, family meals, family nutrition, home cooking, vegetable intakes

## Abstract

Cooking at home is a key recommendation to improve family nutrition. However, parents of young children face barriers to preparing healthy meals. Commercial meal kit subscription services (MKSSs) may support parents to overcome challenges and influence food literacy. This study explored how parents of young children (2–5 years) use MKSSs, examining how food literacy is supported and can be strengthened to promote healthy family meal provisioning. Twenty-five primary meal providers were recruited via social media (e.g. Facebook) for semi-structured interviews over phone or Zoom. Interviews were conducted with participants who had recently purchased meal kits from Australian MKSSs. Transcripts were analysed using inductive thematic analysis followed by deductive mapping to a food literacy framework consisting of four domains (plan and manage, select, prepare, and eat). Three themes captured participants’ meal kit use: (i) managing the complexities of feeding a family with young children, (ii) shifting the mental load, and (iii) broadening culinary horizons for all. Mapping of each theme to the four food literacy domains highlighted that meal kits primarily supported the ‘plan and manage’ and ‘prepare’ domains of family meal provisioning by alleviating pressures in the feeding of young children. The ‘select’ and ‘eat’ domains were less supported. These findings suggest while meal kits may support aspects of food literacy, further guidance on selecting nutritious meals, modifying recipes, involving children, and fostering mealtime practices could strengthen their impact on healthy family meal provisioning. Further research is needed to explore how meal kits could be leveraged to improve parental food literacy and family nutrition.

Contribution to Health Promotion

- Provides new insights regarding how families with young children (2–5 years) use meal kit subscription services in Australia.
- Applies a food literacy framework to identify how meal kits may support parents to plan and manage, select, prepare and eat healthy family meals.
- Identifies opportunities for improvement to help parents select nutritious meals, adapt recipes for health, involve children and promote healthy eating behaviours.

## INTRODUCTION

A nutritious and varied diet is important for health, especially during the formative years of life (0–5 years) for growth and development (World Health Organisation 2003). Globally, adults and children consume sub-optimal diets characterised by low intakes of vegetables (Kalmpourtzidou *et al*. 2020) and wholegrains (Micha *et al*. 2015), and excess intakes of ultra-processed foods (i.e. high in sugar, fat, and salt) (Baker *et al*. 2020) which have been associated with diet-related diseases (Aune *et al*. 2017, Dai *et al*. 2024). This underscores the importance of improving population nutrition, especially in the early years when fundamental eating habits and food preferences are formed, and likely to track into adulthood (Mikkilä *et al*. 2005).

‘Cooking at home’ is a popular public health nutrition strategy, shown to enhance health outcomes including healthier diet quality in adults and children (Mills *et al*. 2017a, Fertig *et al*. 2019) due to higher control over the types of ingredients used, cooking techniques (e.g. oven baked vs. frying) and amount of sugar and salt added during preparation. Moreover, home cooking provides opportunities for children to be exposed to a variety of foods and food behaviours from an early age, which is important for developing lifelong healthy food preferences and eating behaviours (World Health Organisation 2003). As such, ‘cooking at home’ is recognized in the dietary guidelines of Brazil, Canada and France (FAO 2023). However, ‘cooking at home’ is associated with perceived and real barriers, especially for families with young children (5 years and younger). Frequently reported barriers include time and economic constraints, lack of support, low motivation and competing family food preferences (Fulkerson *et al*. 2011, Nepper and Chai 2016, Middleton *et al*. 2020). Families with young children often face additional barriers associated with children’s eating development such as food refusal, and timing meals around child sleep routines (Dwyer *et al*. 2008, Tartaglia *et al*. 2021). As a result of these challenges, among others, many families report using pre-prepared convenience foods or take-away foods (Horning *et al*. 2017, Moran *et al*. 2019).

Cooking is a complex behaviour (Mills *et al*. 2017b), and food literacy has emerged as a useful lens to understand the barriers and diverse knowledge, skills, capabilities and behaviours required to cook at home and meet nutritional needs (Vidgen and Gallegos 2014, Cullen *et al*. 2015, Slater *et al*. 2018). In their conceptual framework, Vidgen and Gallegos (2014) propose that food literacy encompasses four inter-related domains (i.e. plan and manage, select, prepare, and eat) and eleven components that provide the necessary scaffolding to ‘empower individuals, households and communities or nations to protect diet quality through change and strengthen dietary resilience over time’. Through this lens, key aspects of ‘cooking at home’ could be considered to include the knowledge, skills and resources to plan and manage time and budgets, to select foods based on nutritional quality, to prepare foods with/out a recipe while applying safe food hygiene practices, and to eat in a way that promotes health.

Previous research suggests that enhancing parental food literacy may support families in overcoming barriers to family food provisioning (e.g. reduced time, improved taste preferences, and increased knowledge) (Wijayaratne *et al*. 2018). Moreover, higher levels of food literacy have been associated with improved family dietary quality (Wijayaratne *et al*. 2018), including reduced consumption of ultra-processed foods among children at evening meals (‘dinner’) (Martins *et al*. 2020). Therefore, exploring opportunities to improve parental food literacy and support families with young children to cook at home more often is warranted. Especially given the additional barriers to feeding young children, along with the fact that parents are a primary influence on the development of young children’s eating behaviours (Rosenkranz and Dzewaltowski 2008) and food literacy (Vidgen 2016).

Targeting a key barrier of time constraints, commercial meal kit subscription services (MKSSs) have emerged as an option for home-cooked meals, with a large market penetration and widespread global reach (Fernandez and Raine 2021). Within Australia, the MKSS market has evolved rapidly with many providers offering culturally diverse and varied weekly menus to appeal to a range of budgets and preferences (e.g. lower- and higher-cost providers, and vegetarian/plant-based) (Fraser *et al*. 2023a). Using digital ordering platforms and delivery of ‘meal kits’ (i.e. premeasured/semiprepared ingredients and recipes), these services offer convenience and time efficiencies associated with ‘what to cook’ for dinner (Fraser *et al*. 2021, Vos *et al*. 2024).

MKSSs are strongly marketed as convenient and healthy alternatives to take-away/fast food, with meals generally perceived to be healthy and promote increased vegetable consumption among families (Hertz and Halkier 2017, Fraser *et al*. 2021, Vos *et al.* 2024). Several nutrition analyses have suggested that some MKSS meal choices may support vegetable intakes (Moores *et al*. 2020, Fraser *et al*. 2021), though concerns have been raised about the sodium, fat, energy, and vegetable content across meals (Gibson and Partridge 2019, Moores *et al*. 2020, Fraser *et al*. 2021, Nixon and Ensaff 2024). Notwithstanding, there is growing evidence that MKSSs may provide opportunities to support food literacy development and positively influence family diet quality (Gibson and Partridge 2019, Moores *et al*. 2020, Fraser *et al*. 2021, 2023a, 2023b). A recent study examined the theoretical potential of nine Australian MKSSs to influence parental food literacy (Fraser *et al*. 2023b). Thirty-five unique behaviour change techniques (BCTs) (ranging from 19 to 29 BCTs per MKSS) were identified targeting food literacy-related behaviours by reducing barriers related to opportunity (i.e. lack of time/support) and motivation (e.g. low-energy and meal inspiration) by restructuring the home environment (i.e. delivery of meal kit) and enabling cooking at home (Fraser *et al*. 2023b). These findings suggest that MKSSs may positively influence parental food literacy. However, that study assessed the potential of MKSSs using theoretical frameworks, without exploring how families use meal kits in their everyday lives. Therefore, this qualitative study aimed to explore how parents of young children (2–5 years) use MKSSs, to understand which food literacy domains are supported by MKSSs in family meal provisioning and identify opportunities for improving parental food literacy.

## METHODS

### Methodology

This qualitative study was situated within an interpretivist paradigm underpinned by a social constructivist framework. Methodologically, a social constructivist framework acknowledges that participants’ experiences and views are socially constructed (Creswell 2017) and positions the researcher as an active participant in the construction and interpretation of their lived experiences (Liamputtong 2017). Consistent with these epistemological assumptions, the principles of reflexive thematic analysis (Braun and Clarke 2019) were used to explore participants’ experiences and generate rich interpretive themes constructed through the researchers’ subjective and reflexive engagement with the data. Acknowledging researcher positionality and reflexivity is essential to an interpretive methodological approach using reflexive thematic analysis. The research team included three academics (A.S., K.J.C., P.L.) with backgrounds in nutrition, public health promotion and dietetics, particularly early years nutrition. K.F. has a background in health promotion. All authors have qualitative research experience. All authors are parents, with K.F.’s children being in the age range of the study target population at the time of the study. K.F. had used MKSSs intermittently for the past 5 years. To maintain methodological coherence and promote reflexive transparency in the reporting of this study, the Reflexive Thematic Analysis Reporting Guidelines were consulted (Braun and Clarke 2024a). Ethical approval for this study was obtained through Deakin University (HEAG-H 87_2023).

### Recruitment of participants

Eligible participants were Australian parents with at least one child aged 2–5 years, with no children older than 12 years, reflecting the study’s focus on families with young children. To ensure sufficient exposure to MKSSs, eligible participants were required to have purchased more than four meal kits in the past 6 months and self-identify in the screening survey as the person primarily responsible for preparing these meals within their household. Participants were recruited using purposive sampling methods via targeted social media (e.g. research institute led social media and meal kit specific Facebook groups), and snowballing techniques [i.e. researcher (K.F.) personal networks, including early childhood education settings, and participant referral]. Targeted recruitment towards fathers through social media advertisements was also conducted due to a lack of paternal representation in previous research (Davison *et al*. 2018). In line with the principles of reflexive thematic analysis (Braun and Clarke 2021, 2024b), sample size was influenced by practical considerations, including recruitment feasibility and project timelines. The number of participants reflected a ranged of experiences that provided meaningful insights relevant to the research aim. Participants were directed to an online Qualtrics (Qualtrics 2005) screening survey to assess eligibility, complete a demographic questionnaire and provide informed consent. Demographic data included gender, age, country of birth, geographical location, education, employment status, marital status, household income, and age and number of children. Eligible participants were contacted by K.F. to schedule a Zoom or telephone interview.

### Data collection

Semistructured interviews were conducted with parents to generate insights into how families use commercial meal kits. The interview guide was informed by Vidgen and Gallegos (2014) food literacy domains and included questions related to meal kit use compared to general meal provisioning. Furthermore, parents’ preferences for MKSS recipe layout/characteristics to support healthy family meal provisioning were probed. Prior to study commencement, the interview guide was pilot tested with two university students familiar with MKSSs, one of whom is a parent. Slight modifications were made to the interview guide to improve clarity (Supplementary File S1). Interviews were conducted by K.F. between July 2023 and March 2024 via phone and Zoom (Zoom Video Communications 2021). Interviews ran for 30–60 min.

### Data analysis

Interviews were audio recorded and transcribed verbatim using Microsoft Word 2010. Transcriptions were de-identified, checked by the interviewer (K.F.) and imported into NVivo (Lumivero 2023) for analysis. Transcripts were analysed using a combination of inductive and deductive reflexive thematic analysis (Braun and Clarke 2019). Initially, K.F. became familiar with the data through an iterative process of listening to recordings and reading transcripts multiple times. Analytical notes were made based on initial insights of individual interviews and across participants. Transcripts were coded using both semantic (i.e. descriptive labels) and latent (i.e. identifying implicit meaning) coding to address the food literacy domains (deductive) and overarching research aim (inductive). Coding involved a flexible, recursive process of reading, reflecting and questioning by K.F. to develop initial themes. The research team (A.S., K.J.C., and P.L.) each reviewed two to three transcripts and participated in group discussions to share reflections and guide theme formation. These discussions were integral to refinement of theme development. During data analysis, the Vidgen and Gallegos (2014) food literacy framework was applied as a lens to interpret the data. The food literacy framework was applied deductively by mapping each theme to the four domains.

## RESULTS

Fifty-nine individuals completed the online screening survey. Of these, 26 were ineligible (e.g. had purchased fewer than four meal kit boxes in the past 6 months, or had children outside the eligible age range), two did not provide consent, and six could not be contacted. A total of 25 parents (24 mothers and 1 father) participated in interviews (Table 1). The majority of participants were born in Australia (84%), 30–39 years old (64%), married (80%), had two children (52%), and had completed a bachelor’s degree or higher (60%), with household incomes greater than the median Australian household income (∼$92 000) (ABS 2023). Three-quarters (76%) of participants had used MKSSs for more than 12 months (ranging from 2.5 months—7 years). Thirty-two percent had used one MKSS, while 48% had tried three to four different MKSSs based on pricing. Half of participants (48%) regularly ordered meal kits every 1–2 weeks.

**Table 1.** Participant socio-demographic characteristics and meal kit subscription service use (*n* = 25).

Socio-demographic characteristics	Number of participants (*n* = 25)	
	*n*	(%)
**Sex**		
Female	24	96%
Male	1	4%
**Country of origin**		
Australia	21	84%
Other	4	16%
**Age**		
20–29	3	12%
30–39	16	64%
40–49	6	24%
**Marital status**		
Married/living with partner	20	80%
Separate or single (never married)	5	20%
**Qualifications**		
Trade/apprenticeship, Certificate/diploma	10	40%
Bachelor degree or higher	15	60%
**Employment**		
Maternity leave or not employed	4	16%
Casual or studying	3	12%
Part-time	10	40%
Full-time	8	32%
**Annual household income**		
<$100 000	8	32%
$100 000-$200 000	13	52%
>$200 000	4	16%
**Number of children**		
1	8	32%
2	13	52%
3	4	16%
**Age of other children**		
0–5 years	12	48%
>5–12 years	5	20%
**Meal kit subscription service use**		
HelloFresh	15	60%
Marley Spoon	9	36%
Quite Like	1	4%
EveryPlate	14	56%
Dinnerly	12	45%
**Number of meal kits tried/used**		
1	8	32%
2	4	16%
3	6	24%
4+	7	28%
**Frequency of meal kit order**		
Weekly	9	36%
Fortnightly	3	12%
On/off	13	52%
**Duration of meal kit usage**		
<6 months	4	16%
6–24 months	8	32%
>24 months	13	52%

While participants chose to use meal kits for similar reasons (e.g. convenience), when and how they engaged with these was informed by individual family situations. Participant engagement with meal kits revealed three key themes—‘Managing the complexities of feeding a family with young children; Shifting the mental load; and Broadening culinary horizons for all’. Applying Vidgen and Gallegos (2014) food literacy framework to each theme identified how the use of meal kits may support food literacy and also domains that could be targeted to enhance food literacy. Only the first theme mapped to all four food literacy domains, while the remaining themes mapped to the ‘plan and manage’, ‘select’, and ‘prepare’ domains. Table 2 provides a summary of how each theme, as a whole, maps to the domains.

**Table 2.** Mapping of themes to the four domains of Vidgen and Gallegos (2014) food literacy framework.

Food literacy domains	Themes		
	Managing the complexities of feeding a family with young children	Shifting the mental load	Broadening culinary horizons for all
Plan and manage 1.1. Prioritise money and time for food 1.2. Plan food intake (formally and informally) so that food can be regularly accessed through some source, irrespective of changes in circumstances or environment 1.3. Make feasible food decisions which balance food needs (e.g. nutrition, taste, hunger) with available food resources (e.g. time, money, skills, equipment)	Meal kits were used to gain control over demanding roles and responsibilities of feeding a family. Used to navigate busy periods in life and burden of being primary meal preparer	Meal kits were used to reduce decision-making particularly ‘what to cook’—but this is very influenced by costs leading to cyclic usage	Expanding family dietary variety (e.g. types of foods and cuisines); save recipes for later use; reducing planning for special occasions and/or entertaining guests
Select 2.1. Access food through multiple sources and know the advantages and disadvantages of these 2.2. Determine what is in a food product, where it came from, how to store it and use it 2.3. Judge the quality of food	Meal kits allowed parents to accommodate adult and child preferences with little/no extra food work required. Mostly selected meals to suit children's preferences	Decision-making influenced by value for money and time, and nutritional value to a lesser extent. Perception of meals as healthy, nutritious and portion-controlled	Increased confidence to select unfamiliar meals as recipes easy to follow; for some, increased confidence to select meals outside children's taste preferences
Prepare 3.1. Make a good tasting meal from whatever food is available. This includes being able to prepare commonly available foods, efficiently use common pieces of kitchen equipment and having a sufficient repertoire of skills to adapt recipes (written or unwritten) to experiment with food and ingredients 3.2. Apply basic principles of safe food hygiene and handling	Meal kits felt easy to prepare as evenings are a busy time. Low involvement of children in the process dependent on a range of emotional, mental and physical factors	Step-by-step instructions and images reduce perceived cognitive effort during ‘busy’ time of day	Confidence to try new cooking skills as recipes easy to follow; promotes preparation of unfamiliar ingredients
Eat 4.1. Understand food has an impact of personal wellbeing 4.2. Demonstrate self-awareness of the need to personally balance food intake. This includes knowing what foods to include for good health, foods to restrict for good health and appropriate portion size and frequency 4.3. Join in and eat in a social way	What and how meal kit meals were provided to children was shaped by prior beliefs regarding children's taste preference development, eating behaviours and mealtime experiences	None reported	None reported

### Managing the complexities of feeding a family with young children

Participants discussed the complexity and implicit challenges faced when feeding a young family, particularly when balancing the demands of work, study, or raising multiple children. Evenings were described as busy and stressful, with many families desiring quick, easy or fuss-free meals, requiring little cognitive effort. Most participants, regardless of gender or family composition, described meal provisioning as a burden, with meal kits offering a way to manage food-related tasks across the ‘four’ food literacy domains.

Meal kits facilitated forward planning with many participants ordering meal kits during times of uncertainty such as the birth of children and during Covid-19 lockdowns, as well as to manage the everyday stress of raising young children. One participant reported using meal kits pragmatically to manage the challenges of their unique family situation (i.e. a dual-working household, with a shift-working spouse) by planning and selecting meals that could be prepared simultaneously, frozen and reheated at dinnertime to reduce time required for cooking.

Mondays is like my meal prep day. I will just go as crazy as I can to get as much of it done as possible. Last time..I ordered six meals, four portions [each]…I spent the entire day doing it, fitting it in between other things…having it straight away…it's always gonna be nicer than if you've reheated it, but I guess it's gotta be what's practical in your household. **MK015** (Female, dual-parent family, 4 year old child)

A few participants reported sharing meal-related responsibilities (planning, selection and preparation) with their spouse, however, tasks were often unequally divided and dependent on work/activity schedules or children’s needs. For the majority of participants, meal kits were not ordered for the purpose of sharing these tasks but may have facilitated greater involvement of partners/spouses in some families.

Since getting the meal kits, my husband's kind of involved every now and then…I think he just finds the recipe cards really easy to follow…And…he feels like he's helping as well. **MK006** (Female, dual-parent family, 4 and 6 year old children)

Participants’ experiences and beliefs regarding child food preferences and potential for food refusal influenced the selection, preparation and consumption of meals including mealtime routines (e.g. timing and foods offered).

Choosing things that they'll eat is the number one, because if I get something that's more what the adults will eat, it means we'll have to cook something separate for the kids, and I don't want to do that. **MK010** (Female, dual-parent family, 3 and 5 year old children)

Many participants selected meals that their children might eat, opting for meals with familiar components (e.g. meat and vegetables, pasta/rice), or those that could be easily adapted during preparation (e.g. omitting chilli) and serving (e.g. adding preferred raw vegetables instead of salad). A few participants ordered meals based on adults’ taste preferences, preparing separate meals for their children (e.g. nuggets and pasta). In these instances, meal kits primarily ensured adults were consuming ‘healthy’ home-cooked meals instead of relying on take-away foods. Additionally, several participants planned and ordered extra meals to accommodate adult lunches on workdays.

To be honest, usually I don't give him the same meal because I don't think he would eat it…I try to choose meals that are kind of healthier…and I'm not eating as much takeaway and saving money. **MK005** (Female, single parent family, 3 year old child)

Several participants reported preparing meals or components of meals (e.g. cutting vegetables and marinating protein) in advance to reduce time and effort at dinnertime when children required greater attention (e.g. higher emotional needs, tiredness, or bathtime).

I find it a little bit hectic trying to juggle the kids whilst I'm cooking…if I am organised, I'll try and at least get the meal prep done for the evening before my 4 year old comes home. **MK002** (Female, dual-headed family, 1 and 4 year old children)

Involving children in meal preparation was dependent on perceived time and mental capacity. Many participants had concerns about time commitments, safety and mess or felt their children were too young to participate. Preparing meals when children were in daycare/school was preferred by some, while others reported involving children in simple tasks (e.g. mixing) when both parent and child were in the right mood. For some families, meal kits facilitated children’s food-related interactions through brightly coloured recipe cards and the excitement of delivery and unpacking, which increased their willingness to try new/unfamiliar foods.

It's familiarising them with what's coming…they've seen it and they get excited about it and it's come in a box. And they've helped with it. Yeah, they would be more likely to have a go. **MK010** (Female, dual-parent family, 3 and 5 year old children)

### Shifting the mental load

All participants reported that not having to think about ‘what’s for dinner’ was a major driver for using meal kits. Meal kits were perceived to reduce the cognitive (i.e. thinking, planning, and organizing) and emotional (i.e. caregiving, anticipating needs, worry) work experienced when ‘planning’, ‘selecting’, and ‘preparing’ family meals. The convenience of ordering from a set menu and having ingredients delivered was perceived as freeing up mental space, time and energy, especially during the busy evening period.

So that's probably what keeps me coming back…Not having to worry about what I'm going to cook or stressing out because I forgot to take the meat out to thaw… or we've run out of things and having to go to the supermarket to get dinner for the night…it's probably just the convenience. I can literally get a box delivered and unpack five nights worth of dinner in…5 minutes… And that's the hassle out of the way. **MK022** (Female, dual-parent family, 1 and 3 year old children)

While participants perceived meal kits to reduce mental load pressures, value for money was a priority for most participants, who reported multiple strategies to offset meal kit costs. These included only purchasing meal kits when heavily discounted (e.g. 20%–40% off), receiving credit for friend referrals, purchasing the largest meal plan when discounted (i.e. five meals for six people), purchasing the cheapest meal plan (i.e. three meals for two people) and stretching it to feed a family of four or more, and switching between MKSSs to benefit from different company discounts offered. Using these strategies, many participants perceived meal kits to be cheaper than buying groceries at the supermarket. Planning for and anticipating discounts encouraged the regular use of meal kits (e.g. fortnightly, once per month, or 2–3 months), despite some reports of poor-quality ingredients, delivery issues or lack of variety on some menus.

I've been off [MKSS name] because they just stopped having any variety…then I went back to them…because they offered me 40% off. So I took it. **MK016** (Female, dual-headed family, 2 year old child)

Participants described meal kits as easing the mental load when it came to planning, selecting and preparing ‘healthy’ family meals. Most meal kit meals were considered healthy because participants identified that they contained essential macronutrients (e.g. protein and carbohydrates) or vegetables. However, many families avoided selecting vegetarian or plant-based meals due to concerns about children’s food preferences or value for money. Some participants reported adding extra vegetables when preparing meals but this was primarily to bulk up meals or reduce household food waste, rather than to enhance nutritional value. For some, meal kits enabled the family to eat seasonal vegetables without having to consciously think about and shop for ‘in season’ varieties.

I'm finding that just juggling motherhood and then work, it really uses up all of my brain space…I know that the priorities are to eat well and…that takes a lot of thought, so I kind of just been wanting to outsource it a little bit. **MK002** (Female, dual-headed family, 1 and 4 year old children)

Although a few families discussed the high calorie content of certain meal kit meals, these were still considered to be better than the alternative (e.g. take-away or ready-to-heat convenience meals) because they involve cooking from scratch. Almost all participants considered meal kits to be a credible source of nutritionally balanced and portion-controlled meals. Some participants seemed cautiously optimistic that meal kit meals were healthy, suggesting a shift in cognitive responsibility for selecting healthy family meals.

I don't think they're unhealthy. Yeah, wouldn't say they're super healthy. But it’s better than takeaway, I would imagine. I hope. **MK028** (Female, dual-parent family, 2 year old child)

The desire for healthy home-cooked meals requiring little cognitive effort was also reflected in how participants prepared meals. Almost all participants followed the recipes rigidly and reported enjoying the taste of MKSS-branded spices, seasonings and condiments, with some participants purchasing them directly from MKSSs’ marketplace. Only one participant expressed modifying these ingredients when making meal kit meals based on their knowledge of the health impacts of added sugar and salt. The step-by-step recipes, especially those with accompanying images, were seen as valuable in shifting the mental load, especially for self-confessed ‘non-confident’ cooks.

Because you're using a meal kit service, your brain…switches off…So that means even though I know how to cook things from scratch…the whole point is to kind of save me some brain power. **MK029** (Male, dual-parent family, 7 month and 2 year old children)

### Broadening culinary horizons for all

Many participants expressed that meal kits facilitated ‘planning’, ‘selecting’, and ‘preparing’ a wider variety of foods for their family. Recipe cards frequently inspired meal planning and helped break up the ‘cooking rut’, with many participants recreating meals or components such as sauces or side salads.

I think it's…the exposure to other meals that we probably would have never cooked before, or never thought about cooking before. And then sometimes if we're not doing the meal kits, I can take some elements [of the recipe]…and make something else out of it. **MK007** (Female, dual-parent family, 5 and 8 year old children)

Meal kit recipes were described as simple and easy to follow, giving participants confidence to select meals outside their normal repertoire (i.e. new/unfamiliar ingredients or cuisines). For some participants, meal kits increased their confidence in selecting meals outside their children’s comfort zone.

I had no idea what to feed a little kid…And so this just kind of gave me the ideas…I'll pick two that I know he's super comfortable with and maybe the fish he's never tried before and something else. And so it makes me take the risk of trying new flavours or trying new ingredients. **MK013** (Female, single parent family, 5 year old child)

During meal preparation, all participants, especially those with limited cooking skills or experience, reported learning new techniques or enhancing their cooking abilities.

Risotto, I thought that it was out of my league. I’ve never, never, ever cooked it. And I decided to try it one week and…we enjoyed it. **MK019** (Female, single parent family, 18 month old, 3 and 12 year old children)

Even self-described ‘good’ and ‘experienced’ cooks discovered new flavour-pairing methods. For some, this boosted their self-efficacy and confidence to cook a wider range of foods and cuisines in which they had no prior experience (i.e. in childhood). Several participants reported changes in their shopping and cooking behaviours, with many subsequently purchasing and preparing vegetables (e.g. beetroot, cabbage, and Asian Greens) outside their normal repertoire. In these instances, meal kits were perceived to have a positive influence on both adults’ and children’s exposure to a greater variety of foods.

my eldest has definitely started eating a lot more cabbage…he just eats it raw. So now he takes it to school as well…it's not something I would normally buy. Even now, without meal kits, I'm buying…half a cabbage. **MK004** (Female, dual-parent family, 3 and 5 year old children)

### Food literacy mapping

Only the ‘Managing the complexities of feeding a family with young children’ theme mapped to all four domains (Table 2). The ‘eat’ domain was not apparent across the ‘Shifting the mental load’ or ‘Broadening culinary horizons for all’ themes.

## DISCUSSION

This study explored how families with young children (2–5 years of age) use meal kits, to identify opportunities to improve parental food literacy across the four domains (plan and manage, select, prepare, and eat) to support healthy family meal provisioning. Our findings highlight that meal kits mainly support the meal planning and preparation aspects of food literacy, while opportunities exist for meal kits to better promote healthy meal selection and to support healthy eating practices and mealtime routines. This study has important implications for enhancing the use of meal kits to support food literacy and healthy meal provisioning among families.

Despite covering only three to five evening meals per week, meal kits provided participants a sense of control and structure around family meal provisioning, especially when feeding young children. Meal kit use was a considered decision, shaped by each family’s unique situation, including factors such as budgets, time, energy, children’s taste preferences and desire for convenient yet healthy meals. Participants frequently reported that the variety of recipes offered inspiration, new ingredients and cuisines, and opportunities to experience unfamiliar flavours from their own childhood (Spence *et al*. 2016). While parents often perceived meal kits as healthy, they were less likely to cite nutritional quality and the influence on mealtime environment (e.g. time and location, family presence, and interactions during meals) as key benefits or reasons for choosing meals. Engagement with meal kits highlighted an interdependence between the food literacy domains (Vidgen and Gallegos 2014), where decisions regarding one domain often shaped others. For example, financial considerations during ‘planning’ impacted ‘selection’ of meal kit recipes.

A novel finding of this study was that while participants described meal kits as easing the burden of food provisioning, applying the food literacy framework revealed that the load was shifted rather than eliminated. Previous qualitative research with primary meal providers (Fraser *et al*. 2021, Vos *et al.* 2024) reported that meal kits reduced food-related decision making; however, those studies explored participants experiences and did not analyse responses beyond parental perceptions. While our participants noted similar benefits, it was apparent that when using meal kits, they continued to apply high-level organizational and planning skills, making multiple decisions related to factors across the food literacy domains (e.g. cost, health, schedules, and food preferences). Moreover, participants often described complex planning and management strategies when using meal kits, leading to cyclic use. This deliberate and well-managed use of MKSSs may explain our participants’ perception of meal kits as a more affordable alternative to grocery shopping, contrasting with prior research on meal kit costs (Moores *et al*. 2020, Fernandez and Raine 2021, McKay 2023, Nixon and Ensaff 2024). These findings suggest that meal kits appeared to shift, rather than reduce, the mental load, redistributing tasks away from the time of the evening meal. The identification that parents found complex advanced planning to reduce mental load underscores the pressures of preparing evening meals, which is a typically challenging time for families with young children (Fulkerson *et al*. 2011, Nepper and Chai 2016, Middleton *et al*. 2020). For non-meal kit users, food literacy intervention strategies could include a focus on ways to redistribute meal tasks across the day/week, and away from evenings (Wijayaratne *et al*. 2021). For example, health promotion strategies that promote weekly meal planning, advance ingredient prep (e.g. dicing vegetables), and bulk cooking or freezing meals. For meal kit users, MKSSs could actively promote planning and management skills by providing prompts that encourage preparing components of meals in advanced to reduce time and effort at dinnertime, or market freezer-friendly meals.

Most participants prioritized their children’s food preferences when selecting meals to avoid negative mealtime experiences (e.g. food refusal) or the need to prepare alternative meals. This aligns with previous research suggesting that young children’s food preferences are a driving factor in parental food-related purchasing and preparation decisions (Russell *et al*. 2015, Dahl *et al*. 2023). Although participants reported that the range of cuisines (e.g. Mexican and Asian) and meal types (e.g. vegetarian and meat-based) broadened family dietary variety, selecting meals based on children’s known food preferences may limit exposure to a wider variety of foods (Birch 1999). If catering to child preferences is a consistent behaviour, this may reduce opportunities for children to become familiar with and accept new foods, particularly vegetables, and reinforce innate taste preferences (acceptance of sweet and avoidance of bitter/sour tastes). During early childhood, these innate preferences can become more pronounced, underscoring the importance of exposing young children to a varied diet to support lifelong healthy eating habits (Birch 1999).

Similar to previous studies (Fraser *et al*. 2021, Vos *et al.* 2024), most meals were assumed to be healthy, portion-controlled, and nutritionally balanced. Several participants placed their trust in the meal kit companies’ due diligence to provide healthy meals, as suggested by the marketing, assuming meals were developed by nutrition professionals. Thus, this part of mental load appeared to be reduced when it came to selecting and preparing recipes. Most participants followed recipes rigidly and only made modifications/substitutions based on family taste preferences. Meal kit branded seasonings and spice mixes were highly valued for their taste and ease in preparing meals.

This trust in MKSSs to provide nutritious meals may be misplaced, as the nutritional quality of meals have been found to vary substantially, particularly the vegetable content (Gibson and Partridge 2019, Moores *et al*. 2020, Fraser *et al*. 2023a), dietary fibre (Gibson and Partridge 2019, Nixon and Ensaff 2024), salt (Gibson and Partridge 2019, Moores *et al*. 2020, Nixon and Ensaff 2024), energy (from fat and/or protein) (Gibson and Partridge 2019, Moores *et al*. 2020, Nixon and Ensaff 2024) and serving sizes (Moores *et al*. 2020). Some parents were keen to be reassured of the healthfulness of their meal purchasing and may consider such information when choosing MKSSs or meals. Several participants justified their meal selections by considering them heathier alternatives to convenience meals or take-away foods. Meal kit services have an opportunity to enhance their reputation as providers of healthy meals by providing more information about the nutritional quality of their meals and increase the proportion of meals that align with dietary guidelines. Additionally, participants’ perception of vegetarian meals as less cost-effective could be addressed by MKSSs offering discounted plant-based meals to encourage greater vegetable consumption and support public health nutrition goals.

Although participants reported that meal kits enhanced cooking skills and confidence, they did not necessarily promote greater shared responsibility of food-related tasks. Qualitative studies in Australia (*n* = 16) (Fraser *et al*. 2021) and Belgium (*n* = 19) (Vos *et al.* 2024) reported that meal kits increased whole-family participation in meal planning, selection and preparation. However, in our study, few participants reported spousal involvement, likely due to our participant sample consisting mostly of women on maternity leave or working part-time, giving them more time at home. One father in our sample expressed feeling similarly burdened as the primary meal provider. However, his role in food-related tasks was based on higher food agency (Trubek *et al*. 2017) (e.g. enjoyment and confidence) and involvement prechildren, echoing previous findings of reasons for fathers’ contribution (Kuswara *et al*. 2023). Studies in Australia also suggest that fathers are less involved in family meal-related tasks, often due to limited time and cooking skills (Jansen *et al*. 2020, Kuswara *et al*. 2023). Given the benefits of meal kits in reducing time constraints and enhancing cooking skills (Fraser *et al*. 2021, Vos *et al.* 2024), they could be marketed or promoted as a way to engage family members in sharing food-related responsibilities.

Few participants in our study actively involved their children in meal planning, selection, or preparation, citing the time, energy, and mental capacity required. Although child food involvement is known to have nutritional benefits such as increased willingness to try new foods including vegetables (Metcalfe and Fiese 2018, Ng *et al*. 2022), many parents expressed concerns about time, safety and the desire to minimize mess. These barriers align with findings from qualitative studies with families of children aged 2–12 years (Fulkerson *et al*. 2011, Olfert *et al*. 2019, Wijayaratne *et al*. 2021, Laila *et al*. 2023), emphasizing the unique considerations involved in engaging children in this age group. Moreover, Vos *et al.* (2024) reported that child involvement in meal kit selection and preparation was more frequent in families with adolescents (aged 13–18 years) compared to those with school-aged children (aged 6–12 years). This suggests that parents with young children, particularly those aged 2–5 years, may require additional support to engage their children in developmentally age-appropriate tasks safely and at feasible times of day (Dean *et al*. 2021). Several participants noted that the brightly coloured recipe cards, and process of unpacking ingredients, created safer, less demanding opportunities for involving children. These experiences are likely to enhance children’s food literacy by fostering familiarity and sensory engagement (i.e. sight, touch, smell, and play) with foods (Ares *et al*. 2024).

Our findings extend earlier research that examined the theoretical potential of meal kits to influence parental food literacy (Fraser *et al*. 2023b), indicating that the ‘select’ and ‘eat’ domains are less supported by these services. Examples of behaviours which could benefit from support under these domains include participants who did not expect their child to try unfamiliar foods, and those who ate separately from their children or used electronic devices (e.g. TVs and screens) to manage mealtimes. This suggests that parents may benefit from support in the ‘select’ and ‘eat’ domains to introduce unfamiliar foods and create supportive eating environments. One approach could involve incorporating evidence-based tips into recipe cards or marketing materials (Vandeweghe *et al*. 2016) to promote healthy eating practices, such as repeated exposure and parental modelling. Beyond the meal kit sector, these strategies could be incorporated into broader parenting resources, nutrition education programmes or public health messaging.

While meal kits as a paid subscription service may be less accessible for low-income or food insecure families (Moores *et al*. 2020, Fernandez and Raine 2021, McKay 2023, Nixon and Ensaff 2024), these findings regarding how families use meal kits may also have practical application in public health initiatives, including food relief organisations (Horning *et al*. 2021, Fraser *et al*. 2023b). Our participants valued the visually appealing step-by-step recipe cards featuring illustrated instructions, which is a strategy that health promotion or food relief organizations could adopt when presenting recipes. Furthermore, our findings indicate that parents value the convenience of having ‘everything in one box’ through MKSSs, highlighting an opportunity for food relief or fresh-food-box organizations to provide ‘whole-of-meal’ ingredients, matched with recipes to reduce cognitive load. The inclusion of ‘tips/hints’ on recipe cards in the form of ingredient substitutions and/or additions may also support families to bulk-up meals and increase nutritional quality and affordability. Future research could investigate these practical applications to assess their feasibility and acceptability.

A major strength of this study is the application of a food literacy framework to understand the intersection with parents’ meal kit usage and identify opportunities to enhance parental food literacy. Moreover, this study offers valuable insights into the experiences of families with preschool-aged children (2–5 years), addressing an under-researched, priority population, in the context of meal kit use. Limitations should also be considered when interpreting our findings. Despite using a targeted advertising strategy to recruit fathers, our study sample consisted primarily of mothers. While this is reflective of current research indicating that mothers are mostly responsible for domestic food-related duties (Fulkerson *et al*. 2011, Fielding-Singh 2017), future research should continue to seek fathers’ inputs. Furthermore, this study was limited by a relatively homogenous sample with most participants born in Australia, university educated, from moderate to high income households and living in dual-parent families. In addition, participant ethnicity and race were not collected during the screening process. Future research should assess whether this reflects meal kit users and seek to recruit more socioeconomically and culturally diverse participants to better capture the experiences of different population groups.

## CONCLUSION

This study offers valuable insights into how families with young children use meal kits to navigate the challenges of feeding young children, and how meal kits may be leveraged to enhance parental food literacy. Meal kits may serve as a valuable tool to help parents plan, manage and prepare meals in advance, alleviating the pressures of family food provisioning, especially during evening meals when children require greater attention. Our findings indicate that the ‘select’ and ‘eat’ food literacy domains are currently less supported by the use of meal kits. MKSSs could potentially deliver more on parents’ expectations of their healthy branding by enhancing their value in feeding young children. This could include aiding in selection and affordability of healthy meals, adapting recipes for health, encouraging children’s involvement in age-appropriate tasks, and fostering mealtime practices that promote healthy eating behaviours. Our study suggests that opportunities exist for meal kits to support parental food literacy and further research is recommended to explore how this may be achieved to optimize family diet quality and mealtimes.

## Supplementary Material

daaf105_Supplementary_Data

## Data Availability

The data underlying this article cannot be shared publicly due to the privacy of individuals that participated in the study. The data will be shared on reasonable request to the corresponding author.
