# Supplementary material for: Can Australian meal kits support food literacy and healthy family meal provisioning? A qualitative study
Source: Health Promot Int. 2025 Jul 8;40(4):daaf105. doi: 10.1093/heapro/daaf105 (PMC12235520; doi:10.1093/heapro/daaf105)
Supplement: daaf105_Supplementary_Data [file daaf105_supplementary_data.docx]

**Introduction / Overview of study**

Thank you again for taking the time to participate.

This study aims to explore how families with young children use meal kits. We are hoping to find out how meal kits are used in the planning, selecting, preparing and eating phases of family meals. We would like to know how they may help or make it difficult for you to feed your family and provide healthy meals, particularly vegetables for your children. We would also like to explore how meal kits could be designed/modified to better support you to provide healthy family meals.

As a reminder, the interview will be recorded but all information provided will remain confidential. Any identifying information about yourself or family members will be removed from the transcript.

You’ll be prompted in a moment to confirm your consent agree to participate in this recorded interview. [press RECORD]

**Icebreakers:**

I see that you’ve indicated that you have XX children. Can you tell me a bit about them, including how old they are? What do they do during the day?

Discuss responses on Qualtrics form i.e. children ages, work arrangements, family composition, location.

| **Interview Topics** | **Example Questions** |
| --- | --- |
| **Role in feeding the family –** | To start with, before we talk about MKs, I’d like to know a little bit about the role you play when it comes to feeding your family particularly around the evening or dinner meal.  *Prompt: Q. Can you tell me about who’s responsible in your house for deciding what to cook, purchasing groceries, preparing and cooking meals?*  Q. How do you feel about doing this role in general?  Q. How do you feel about planning, shopping and cooking for meals? |
| **Meal Kit Usage –** | Let’s talk about your experience with MKs:  Q. I see that you’ve used XY MKSS. Can you tell me about your experience with XY MKSS – when you started, how long you did it for, how many meals you order...  Q. Why did you choose to use MK meals initially?  Q. What influences your decision to use MKs? |
| **Meal Kit Usage – Food Literacy Domains (Vidgen and Gallegos, 2014)** | Explore **who** is involved in each phase and **why**:  Plan and manage:  Q. When planning meals using MK app/website, can you tell me how you go about this process?  *- Prompts: planned weeks in advance*  Q. Are the kids invovled in choosing the meals?  Select:  Q. Tell me about the types of MK meals you generally order.  Q. What influences your meal choices and why?  Q. Do you choose vegetarian meals? Why/why not?  Q. When using MKs, do you consider them a healthy way of feeding the family?  *- Prompts: portion sizes, nutritional content, fresh ingredients*  Q. What types of meals does your family eat on non-MK nights/weeks? Q. What influences these choices?  Preparation:  Q. Who usually prepares the MK meals?  Q. Are the children invovled in unpacking, or preparing the meals?  *- Prompts: touching, talking, looking at raw vegetables?*  Q. Tell me about any modifications/substitutions made to MK recipes? Why? *Prompts: sauces, spices, condiments*  Q. How confident are you in your knowledge or ability to adapt MK recipes?  Q. Who usually prepares meals on non-MK nights/weeks?  Eat:  Q. Tell me about mealtimes in your family on MK nights  Q. How do you portion out the MK meals?  *- Prompts: kids portion? Kids same or deconstructed? Additional ingredients?*  Q. Does this differ for non-MK nights? |
| **Barriers and enablers to using MKs to provide healthy family meals, particularly vegetables.** | Explore **barriers and enablers** to providing healthy meals -  Q. Have you noticed a change in your family’s/children’s diet since using MKs?  *- Prompts: variety, vegetable intakes, fast food*  Q. Have you noticed a change in the way your children eat since using MKs, particularly vegetables?  Q. Which vegetables work or don’t work?  *- Prompts: variety, vegetable intakes, willingness to try*  Explore **barriers and enablers** regarding Food Literacy -  Q. Since using MKs, have you noticed any changes in the way you plan, prepare or cook meals for your family?  *- Prompts: other family members’ skills, knowledge, variety*  Q. What has been your experience using the MK recipe cards/instructions?  Q. What features do you like on MK recipes/cards? Why? |
| **Preferences for MK features and characteristics** | Here are some recipe cards from the different MKSS providers.  Q. Which recipes appeal to you? *Why/why not?*   - Prompts: Layout, colours, spacing, text |

MKSSs = meal kit subscription services, MKs = meal kits

**Anything else that you would like to share about your experience with meal kits?**

Thank you for your time and participation in this study. If you are aware of anyone that may be interested in participating in our study, please feel free to pass on the recruitment flyer or direct them to our Facebook advertisement/IPAN website for further details.

**References**

Vidgen, H. A. and Gallegos, D. (2014) Defining food literacy and its components. *Appetite*, **76**, 50-59. <https://doi.org/10.1016/j.appet.2014.01.010>.
